# Histological chorioamnionitis, antenatal steroids, and neonatal outcomes in very low birth weight infants: A nationwide study

**DOI:** 10.1371/journal.pone.0224450

**Published:** 2019-10-29

**Authors:** Hyun-Seung Lee, So Young Kim

**Affiliations:** 1 Department of Pediatrics, CHA Gangnam Medical Center, CHA University School of Medicine, Seoul, Korea; 2 Department of Pediatrics, College of Medicine, The Catholic University of Korea, Seoul, Korea; Johns Hopkins University, UNITED STATES

## Abstract

**Background:**

The aim of this study was to investigate whether some associations between histological chorioamnionitis (HCA) and favorable neonatal outcomes might be linked to those of antenatal steroids (AS) by determining the separate as well as the combined associations of HCA and AS with neonatal outcomes in very low birth weight infants (VLBWIs).

**Methods:**

This was a population-based study of VLBWIs born at 20–33 weeks’ gestation between January 2013 and December 2015 from the Korean Neonatal Network. A total of 4652 VLBWIs were enrolled for prevalence study. Of these, 2900 singleton VLBWIs were used for outcome analyses to evaluate individual associations of HCA and AS simultaneously with correction for potential perinatal factors and an interaction term of HCA and AS.

**Results:**

The overall prevalence of HCA was 34.9% (1623 VLBWIs). Multivariable logistic regression demonstrated that HCA was associated with decreased mortality (adjusted odds ratio [aOR], 0.51; 95% confidence interval [CI], 0.29–0.91; *P* = 0.022), AS were associated with reduction in mortality (aOR, 0.59; 95% CI, 0.39–0.90; *P* = 0.014) and neonatal seizure (aOR, 0.57; 95% CI, 0.37–0.86; *P* = 0.008), and a combination of HCA and AS was associated with remarkably lowered severe intraventricular hemorrhage by interacting with each other (aOR, 0.47; 95% CI, 0.25–0.88; *P* = 0.019).

**Conclusions:**

We suggest that in VLBWIs HCA and AS may be favorable independent factors for neonatal outcome and may also work in synergy for neuroprotection.

## Introduction

Histological chorioamnionitis (HCA) is defined as an acute inflammation of chorionic and amniotic layers of fetal membranes, many cases of which are clinically silent [1,2]. The placental tissue inflammation can lead to intra-amniotic inflammation and fetal inflammatory response syndrome linked to elevated pro-inflammatory cytokines (interleukins [ILs] -1 and -6 and tumor necrosis factor-α), eventually resulting in fetal injury [3–5]. Prenatal exposure to intrauterine infection/inflammation has been associated with a variety of adverse outcomes, including preterm birth [6], neonatal sepsis [7,8], perinatal mortality [3,9], and neonatal short- and long-term morbidities from fetal brain and lung insults [3–5,8]. However, conflicting data have been found. Recent population-based or large cohort studies of preterm infants after adjusting for perinatal factors including gestational age (GA), have reported the associations between HCA and reduction in adverse neonatal outcomes including mortality [6,10], respiratory distress syndrome (RDS) [11,12], bronchopulmonary dysplasia (BPD) [13,14], late-onset sepsis [15], and patent ductus arteriosus (PDA) [11].

Antenatal steroids (AS) have been accepted as standard medications given to women with imminent preterm parturition because of its effectiveness in preventing newborn outcomes such as RDS, intracranial hemorrhage, necrotizing enterocolitis (NEC), and death [16,17]. Unlike HCA, AS have been consistently considered to produce better prognosis along with greater GA and greater birth weight (BW) as factors that may affect mortality and morbidities of preterm infants [18]. The current guidelines of AS therapy recommend its broad application including cases of sepsis, based on evidence from hospital settings of high income countries [16,17]. Recently, population-based studies of very preterm infants using multivariable modeling and a meta-analysis of preterm cohorts documented that in the setting of HCA, AS were associated with reduced risks for mortality [19–21], RDS [19–21], total [19–21] and severe intraventricular hemorrhage (IVH) [19], neonatal seizure [20], neonatal sepsis [20,21], and PDA [19], but the elevated risk for chronic lung disease [21].

The reported favorable results of HCA in some of neonatal outcomes from the adjusted analyses of large preterm cohorts under high coverage rates of AS have been shown to overlap with those of AS previously published [6,10,12–14]. A systematic review addressed a tendency that the correlations between chorioamnionitis and adverse neurological outcomes were attenuated since the era of widespread use of AS [22]. To date, only a few studies of infants born preterm discussed the possibility that AS might affect outcomes from chorioamnionitis [10,23]. Accordingly, we hypothesized that some associations between HCA and favorable neonatal outcomes among preterm infants would be linked to those of AS. We performed a retrospective observational study using nationwide data on very low birth weight infants (VLBWIs) from the Korean Neonatal Network (KNN). The study aimed to determine the separate as well as the combined associations of HCA and AS with neonatal outcomes in VLBWIs. We first evaluated individual associations of HCA and AS with neonatal outcomes in VLBWIs simultaneously, then, two-step multivariable analyses were performed with correction for potential perinatal factors that may affect neonatal outcomes and an interaction term of HCA and AS.

## Materials and methods

This study was a retrospective analysis of prospectively collected clinical data on VLBWIs (< 1,500 g BW) born at < 34 weeks’ gestation between January 2013 and December 2015 who were admitted to neonatal intensive-care units (NICU) of perinatal centers registered for the KNN [24]. After excluding infants with insufficient placental histopathology data, the first study population was formed for prevalence assessment. Among these, the second study population was selected for final outcome analyses after excluding infants with major congenital malformations, in multiple gestation, or in outborn status.

### Data collection

The KNN is a nationwide database on VLBWIs in NICU hospitalization of up to 60 participating facilities (December 2015) during the inclusion period. This accounts for approximately 70% of overall admissions of VLBWIs in South Korea [24]. The current study was approved by the KNN data management committee. Antenatal, perinatal, and neonatal information were acquired from the KNN database. Infants’ data such as GA, BW, sex, small for gestational age, 1- and 5-min Apgar scores, and delivery mode were collected. Maternal data including age, gravidity, parity, hypertension, diabetes, prolonged rupture of membranes (> 18 hours), and the use of AS were retrieved. The following short-term neonatal outcomes were recorded: mortality, RDS, BPD, seizure, IVH, periventricular leukomalacia (PVL), sepsis, NEC (stage ≥ 2), PDA, and retinopathy of prematurity (ROP).

### Definitions

HCA was defined as the presence of acute inflammatory change of polymorphonuclear leukocyte infiltration in any part of the amnion, chorionic decidua, umbilical cord, and the chorionic plate based on pathologic review of the placenta by pathologists at each participating facility according to the modified Salafia *et al*’ criteria by Yoon *et al* [25,26]. GA was determined by the best obstetric estimate using the date of the last menstrual period and/or ultrasonography. AS exposure was defined as maternal receipt of at least one dose of any corticosteroid during pregnancy. Small for gestational age was defined as a BW less than the 10th percentile for the GA based on the sex-specific growth chart for preterm infants by Fenton [27]. RDS was diagnosed by the presence of clinical respiratory distress and a compatible radiographic appearance, the requirement for invasive or noninvasive mechanical ventilation. BPD was defined as dependency on oxygen supplementation or positive pressure support at 36 weeks of postmenstrual age using the National Institutes of Health Workshop severity-based diagnostic criteria [28]. IVH was defined using Papile’s criteria [29]. The highest grade among all results obtained during hospitalization from brain ultrasonography by the individual policy of each hospital was recorded. PVL included only cystic PVL demonstrated by brain ultrasonography or magnetic resonance imaging during hospitalization. Seizures were diagnosed by the presence of clinical symptoms, including clonic, tonic, and subtle seizure manifestations as a consequence of a paroxysmal alteration in motor, and occasionally autonomic function, and the requirement for anticonvulsants for therapeutic and/or prophylactic purposes. Neonatal sepsis was defined as bacterial or fungal infection documented by a positive blood culture in the first 7 days of life (early-onset sepsis) or thereafter (late-onset sepsis) in the presence of clinical signs of infection [30]. NEC was defined according to Bell’s criteria [31] (stage 2 or greater). PDA referred to the PDA detected by echocardiography and requiring pharmacological treatment for therapeutic and/or prophylactic purposes. ROP was defined according to the International Classification for ROP [32]. The maximum stage of ROP was adopted. Mortality was termed death in the NICU that occurred before the first hospital discharge.

### Statistical analysis

All statistical analyses were performed using SPSS 17.0 software (SPSS, Inc., Chicago, IL). Data are expressed as number (%) or median (interquartile range). Univariable analyses were conducted using χ^2^ test for categorical variables and Mann-Whitney *U* test for continuous variables. Two series of multivariable logistic regression analyses including variables of interest (HCA and AS) were performed with or without an interaction term (HCA × AS). Multivariable models accounted for perinatal characteristics as possible confounder covariates in 2 ways: after excluding 1- and 5-min Apgar scores from the baseline perinatal variables, 1) the seven variables that were significantly different between the HCA+ and HCA–groups and 2) all the eleven variables. Goodness-of-fit Chi-square statistics were ascertained for model fit [33]. A two-tailed *P* < 0.05 was considered statistically significant.

### Ethics statement

This study was approved by the Samsung medical center institutional review board (2013-03-002). Data registration in the KNN was approved by the institutional review board at each participating hospital. Written informed consent was obtained from parents of infants upon enrollment by NICU staff at each hospital. All methods were carried out in accordance with the institutional review board-approved protocol and in compliance with relevant guidelines and regulations.

## Results

### Study populations

**Fig 1:**
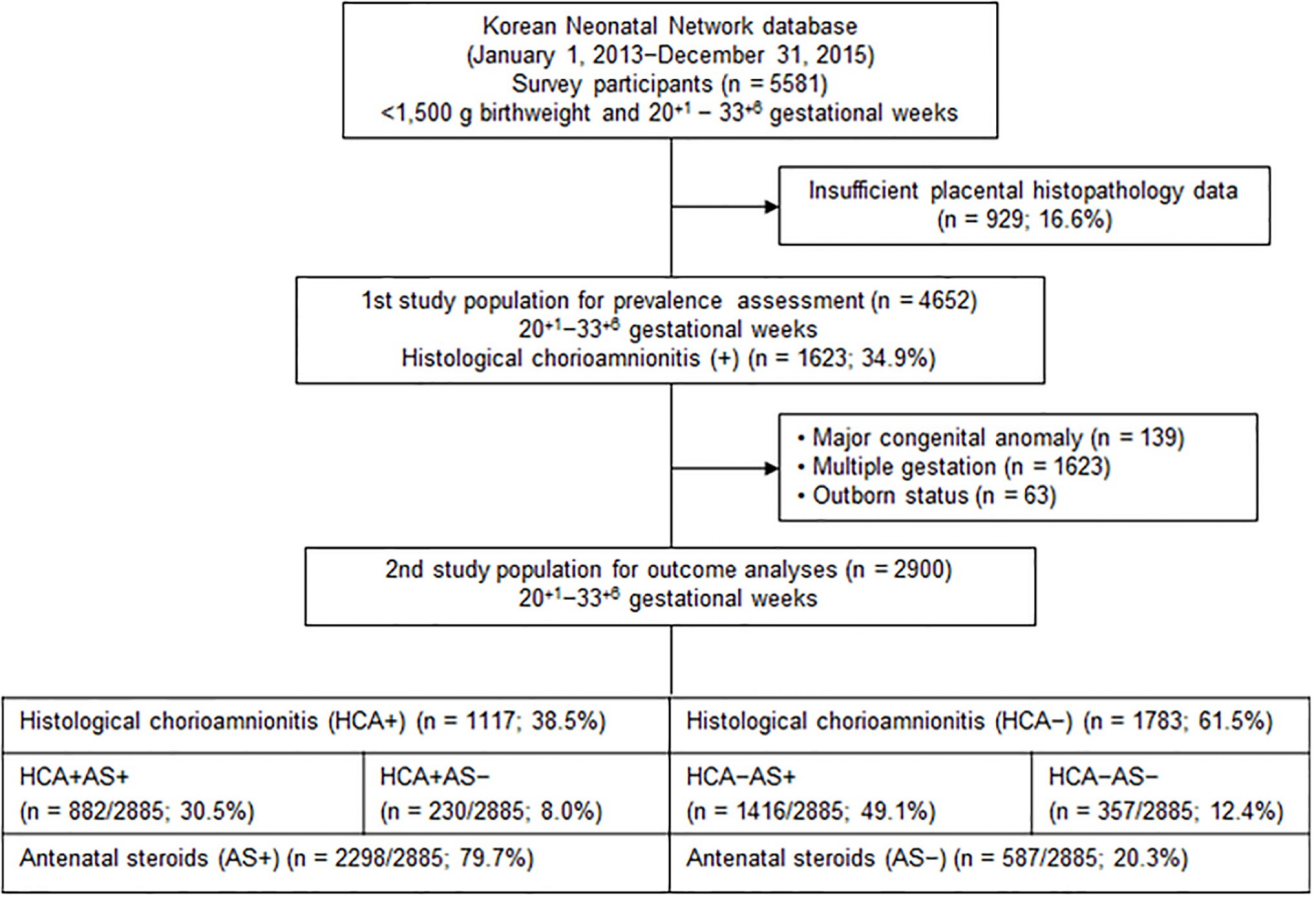
Flow chart of study populations from the Korean Neonatal Network database.

Fig 1 shows a flowchart of study populations. We reviewed a total of 5581 VLBWIs born between 20^+1^ and 33^+6^ weeks of gestation during the study period and registered in the KNN. Among them, 4652 (83.4%) infants born at 20^+1^–33^+6^ weeks’ gestation had available data on pathological examination of the placenta and were included in the first population for prevalence study. Of the 4652 infants, 2900 (62.3%) infants born at gestational ages between 20^+1^ and 33^+6^ weeks were enrolled in the second study population for final outcome analyses after excluding 139 infants with major congenital malformations, 1623 infants from a multiple pregnancy, and 63 infants born outside the participant centers. These 2900 infants were then categorized into HCA+ and HCA–groups depending on the placental pathology of HCA and AS+ and AS–groups according to AS exposure.

### Prevalence of HCA

Among 4652 VLBWIs with available placental histopathology data, 1623 (34.9%) were exposed to maternal HCA (Fig 1).

### Baseline perinatal characteristics

Table 1 shows baseline perinatal characteristics of the second study population. Infants exposed to maternal HCA were younger and lighter at birth, were more likely to have vaginal birth, had lower 1- and 5-min Apgar scores, and had less small for gestational age than those unexposed (all *P*s < 0.001). Infants exposed to AS had higher 1- and 5-min Apgar scores (all *P*s < 0.001) than those unexposed. Mothers with HCA had greater parity (*P* = 0.004), lower rates of maternal hypertension (*P* < 0.001), and higher occurrence of prolonged rupture of membranes (> 18 hours) (*P* < 0.001) than those without HCA. Mothers with AS administration had less gravidity and parity (*P* = 0.013 and *P* < 0.001, respectively) and higher occurrence of prolonged rupture of membranes (> 18 hours) (*P* < 0.001) than those without AS administration. There was no significant difference in the rate of AS exposure between HCA+ and HCA–groups or in the prevalence of HCA between AS+ and AS–groups.

**Table 1:** Demographic perinatal characteristics of 2900 very low birth weight infants according to exposure of maternal HCA and AS.

	HCA+ (n = 1117)	HCA–(n = 1783)	P*	AS+ (n = 2298)	AS–(n = 587)	P*
**Maternal age, years**	33.0 (30.0–35.0)	33.0 (30.0–36.0)	0.970	33.0 (30.0–36.0)	33.0 (30.0–36.0)	0.971
**Gravidity**	2.0 (1.0–3.0)	2.0 (1.0–3.0)	0.069	2.0 (1.0–3.0)	2.0 (1.0–3.0)	0.013
**Parity**	1.0 (0.0–1.0)	0.0 (0.0–1.0)	0.004	0.0 (0.0–1.0)	1.0 (0.0–1.0)	<0.001
**Maternal hypertension, n (%)**	109 (9.8)	639 (35.8)	<0.001	606 (26.4)	139 (23.7)	0.184
**Maternal diabetes, n (%)**	90 (8.1)	138 (7.7)	0.757	180 (7.8)	46 (7.8)	0.998
**ROM > 18 h, n (%)**	458/1089 (42.1)	334/1765(18.9)	<0.001	696/2264 (30.7)	94/576 (16.3)	<0.001
**Caesarean delivery, n (%)**	690 (61.8)	1401 (78.6)	<0.001	1674 (72.8)	406 (69.2)	0.076
**Gestational age, weeks**	27.4 (25.6–29.3)	29.0 (27.0–30.9)	<0.001	28.4 (26.4–30.1)	28.6 (26.0–30.6)	0.965
**Birth weight, g**	1040.0 (800.0–1260.0)	1090.0 (850.0–1310.0)	<0.001	1070.0 (830.0–1280.0)	1070.0 (830.0–1310.0)	0.322
**Male gender, n (%)**	582 (52.1)	904 (50.7)	0.462	1179 (51.3)	297 (50.6)	0.759
**SGA, n (%)**	102 (9.1)	405 (22.7)	<0.001	409 (17.8)	98 (16.7)	0.531
**Apgar score at 1 min**	4.0 (3.0–6.0)	5.0 (3.0–6.0)	<0.001	5.0 (3.0–6.0)	4.0 (2.0–6.0)	<0.001
**Apgar score at 5 min**	7.0 (5.3–8.0)	7.0 (6.0–8.0)	<0.001	7.0 (6.0–8.0)	7.0 (5.0–8.0)	<0.001
**AS, n (%)**	882/1112 (79.3)	1416/1773 (79.9)	0.722			
**HCA, n (%)**				882 (38.4)	230 (39.2)	0.722

Data are presented as numbers of patients (%) or median (interquartile range). HCA, histological chorioamnionitis; AS, antenatal steroids; ROM, rupture of membranes; SGA, small for gestational age.

### HCA, AS, and neonatal outcome: Univariable and multivariable analyses

Relationships of HCA and AS status with neonatal outcomes in the second study population are shown in Tables 2 and 3. Only the multivariable analysis results after accounting for all the baseline perinatal variables except 1- and 5-min Apgar scores are listed in the tables, because the significant outcome variables are identical in the results after adjusting for either the seven baseline perinatal variables significantly different between the HCA+ and HCA–groups or all the baseline perinatal variables except 1- and 5-min Apgar scores. In unadjusted models, HCA was associated with more prevalent neonatal morbidities, including RDS (*P* < 0.001), BPD (all grades and severe grade [both *P*s < 0.001]), total IVH (*P* = 0.007), neonatal seizure (*P* < 0.001), early-onset sepsis (*P* = 0.025), and severe ROP (*P* < 0.001) (Table 2). The use of AS was linked to reduced proportion of mortality (*P* = 0.004), severe IVH (*P* < 0.001), PVL (*P* = 0.045) and neonatal seizure (*P* < 0.001) (Table 2). Multivariable logistic regression without interaction terms after covarying for all the baseline perinatal characteristics except 1- and 5-min Apgar scores revealed that HCA was correlated with reduced incidence of mortality (adjusted odds ratio [aOR], 0.67; 95% confidence interval [CI], 0.50–0.89; *P* = 0.005), severe IVH (aOR, 0.70; 95% CI, 0.52–0.94; *P* = 0.016), cystic PVL (aOR, 0.61; 95% CI, 0.45–0.83; *P* = 0.001), and NEC (stage ≥ 2) (aOR, 0.66; 95% CI, 0.46–0.94; *P* = 0.020) (Table 2). The use of AS was associated with lower occurrence of mortality (aOR, 0.69; 95% CI, 0.50–0.95; *P* = 0.022), RDS (aOR, 0.71; 95% CI, 0.52–0.96; *P* = 0.028), severe IVH (aOR, 0.58; 95% CI, 0.43–0.80; *P* = 0.001) and neonatal seizure (aOR, 0.58; 95% CI, 0.43–0.79; *P* = 0.001) (Table 2). When an interaction term (HCA × AS) was added to multivariable models, the following results were found: the interaction term (HCA × AS) was associated with prominent reduction in severe IVH (aOR, 0.47; 95% CI, 0.25–0.88; P = 0.019) while HCA was associated with decreased mortality (aOR, 0.51; 95% CI, 0.29–0.91; P = 0.022), and the use of AS was associated with reduction in mortality (aOR, 0.59; 95% CI, 0.39–0.90; P = 0.014) and neonatal seizure (aOR, 0.57; 95% CI, 0.37–0.86; P = 0.008) (Table 3). The other outcome variables did not show any significance (the data were not shown in the result section).

**Table 2:** Associations of neonatal outcomes with HCA and AS status.

Outcomes	Test group	N (%)	Reference group	N (%)	*P* (χ^2^)	Adjusted OR^a^ (95% CI)	*P*^a^
**Mortality**	HCA+	150/1036 (14.5)	HCA–	208/1702 (12.2)	0.089	0.67 (0.50–0.89)	0.005
	AS+	262/2169 (12.1)	AS–	93/557 (16.7)	0.004	0.69 (0.50–0.95)	0.022
**RDS**	HCA+	980/1117 (87.7)	HCA–	1444/1783 (81.0)	<0.001	0.98 (0.75–1.29)	0.892
	AS+	1911/2298 (83.2)	AS–	498/587 (84.8)	0.328	0.71 (0.52–0.96)	0.028
**BPD (all grades)**	HCA+	671/978 (68.6)	HCA–	870/1590 (54.7)	<0.001	0.88 (0.68–1.13)	0.323
	AS+	1234/2059 (59.9)	AS–	298/497 (60.0)	0.991	0.74 (0.56–1.00)	0.046
**Severe BPD**	HCA+	240/978 (24.5)	HCA–	249/1590 (15.7)	<0.001	1.24 (0.98–1.59)	0.078
	AS+	389/2059 (18.9)	AS–	98/497 (19.7)	0.674	0.85 (0.64–1.13)	0.252
**IVH (all grades)**	HCA+	511/1083 (47.2)	HCA–	731/1739 (42.0)	0.007	0.88 (0.74–1.05)	0.146
	AS+	974/2254 (43.2)	AS–	258/553 (46.7)	0.144	0.83 (0.68–1.02)	0.080
**IVH (grade ≥ 3)**	HCA+	119/1083 (11.0)	HCA–	160/1739 (9.2)	0.122	0.70 (0.52–0.94)	0.016
	AS+	193/2254 (8.6)	AS–	80/553 (14.5)	<0.001	0.58 (0.43–0.80)	0.001
**PVL**	HCA+	88/1079 (8.2)	HCA–	159/1735 (9.2)	0.358	0.61 (0.45–0.83)	0.001
	AS+	184/2247 (8.2)	AS–	60/552 (10.9)	0.045	0.81 (0.59–1.12)	0.201
**Seizure**	HCA+	131/1117 (11.7)	HCA–	143/1783 (8.0)	<0.001	0.96 (0.72–1.28)	0.798
	AS+	186/2298 (8.1)	AS–	83/587 (14.1)	<0.001	0.58 (0.43–0.79)	0.001
**Early-onset sepsis**	HCA+	75/1114 (6.7)	HCA–	85/1782 (4.8)	0.025	1.03 (0.72–1.48)	0.857
	AS+	119/2294 (5.2)	AS–	39/587 (6.6)	0.167	0.85 (0.57–1.26)	0.417
**Late-onset sepsis**	HCA+	179/1114 (16.1)	HCA–	293/1782 (16.4)	0.791	0.80 (0.63–1.00)	0.050
	AS+	381/2294 (16.6)	AS–	89/587 (15.2)	0.397	1.13 (0.87–1.47)	0.366
**NEC (stage ≥ 2)**	HCA+	63/1107 (5.7)	HCA–	116/1775 (6.5)	0.361	0.66 (0.46–0.94)	0.020
	AS+	143/2288 (6.3)	AS–	34/579 (5.9)	0.736	1.09 (0.73–1.65)	0.667
**PDA (medically managed)**	HCA+	412/1101 (37.4)	HCA–	639/1766 (36.2)	0.504	0.88 (0.74–1.06)	0.170
	AS+	839/2267 (37.0)	AS–	208/585 (35.6)	0.515	1.16 (0.95–1.43)	0.146
**ROP (grade ≥ 3)**	HCA+	155/978 (15.8)	HCA–	153/1558 (9.8)	<0.001	0.91 (0.67–1.23)	0.524
	AS+	247/2035 (12.1)	AS–	59/488 (12.1)	0.977	1.10 (0.76–1.61)	0.613

HCA, histological chorioamnionitis; AS, antenatal steroids. OR, odds ratio; CI, confidence interval; RDS, respiratory distress syndrome; BPD, bronchopulmonary dysplasia; IVH, intraventricular hemorrhage; PVL, cystic periventricular leukomalacia; NEC, necrotizing enterocolitis; PDA, patent ductus arteriosus (medically managed); ROP, retinopathy of prematurity.

^a^Adjusted values were obtained from multivariable logistic regression models without interaction terms including the following variables: HCA, AS, maternal age, gravidity, parity, maternal hypertension, maternal diabetes, rupture of membranes > 18 h, caesarean delivery, gestational age, birth weight, infant sex, and small for gestational age.

**Table 3:** Development of severe IVH, mortality, and neonatal seizure.

Outcomes	Variables	Adjusted OR^a^ (95% CI)	*P*^a^
**IVH (grade ≥ 3)**	HCA	1.21 (0.71–2.09)	0.484
	AS	0.82 (0.53–1.26)	0.362
	HCA × AS	0.47 (0.25–0.88)	0.019
**Mortality**	HCA	0.51 (0.29–0.91)	0.022
	AS	0.59 (0.39–0.90)	0.014
	HCA × AS	1.41 (0.75–2.65)	0.292
**Seizure**	HCA	0.93 (0.55–1.57)	0.782
	AS	0.57 (0.37–0.86)	0.008
	HCA × AS	1.05 (0.57–1.93)	0.870

IVH, intraventricular hemorrhage; HCA, histological chorioamnionitis; AS, antenatal steroids. OR, odds ratio; CI, confidence interval.

^a^Adjusted values were obtained from multivariable logistic regression models with an interaction term of HCA and AS including the following variables: HCA, AS, HCA × AS, maternal age, gravidity, parity, maternal hypertension, maternal diabetes, rupture of membranes > 18 h, caesarean delivery, gestational age, birth weight, infant sex, and small for gestational age.

## Discussion

The current study of VLBWIs demonstrated that the higher risk of HCA for some adverse neonatal outcomes (RDS, total and severe BPD, total IVH, neonatal seizure, early-onset sepsis, and severe ROP) shown in unadjusted analyses was not significant any longer in multivariable logistic regression analyses (Table 2). In the multivariable models, the associations of HCA with reduction in severe IVH, cystic PVL, and NEC were novel findings that were not found in prior reports. The multivariable models also showed the association of AS with reduced severe IVH. Protective effects of AS for IVH and NEC in preterm infants have been previously reported [16,17]. In addition, most recent adjusted data, particularly those with large preterm cohorts, have indicated no effect of chorioamnionitis on neurological outcomes [22,34]. Therefore, to find out clearer associations between HCA and neonatal outcomes, we explored the interaction between HCA and AS. After controlling for all the baseline perinatal factors except 1- and 5-min Apgar scores and an interaction term of HCA and AS, the final three major findings that represented HCA and AS as possible favorable factors for neonatal outcome of VLBWIs were found: significant associations 1) between HCA and decreased mortality, 2) between AS and reduction in mortality and neonatal seizure, and 3) between the interaction term of HCA and AS and remarkably lowered severe IVH (Table 3). These results suggest that in VLBWIs HCA and AS may have not only individual advantages but also a combined benefit by working in synergy.

To date, a variety of underlying mechanisms have been suggested to explain the advantages of AS therapy for preterm birth. These include acceleration of fetal organ maturation [16], cerebral capillary stabilization [35], more stable blood-pressure hemodynamics [36], basal metabolism reduction that inhibits cerebral hypoperfusion-mediated injury [37], and organotoxic cytokine suppression [38]. These may explain the associations of AS with neuroprotection in VLBWIs shown in our analyses, consistent with previous reports [16,17,19–21].

In contrast, studies on roles of HCA in premature infants have been largely focused on the harmful effects of intrauterine infection/inflammation, including cytokine-related organ injury [4,5,39] and impaired lung development via decreased Wingless-Int signaling by intra-amniotic lipopolysaccharide exposure [40]. However, there have been evidences that prenatal inflammation has benefits for preterm neonates by inducing host defenses, including fetal lung maturation via increased endogenous adrenal cortisol [41] and IL-6-induced surfactant protein-A synthesis [42] in preterm infants unexposed to AS and innate immune maturation and tolerance in animal models [43]. Shimoya *et al*. [42] have performed an experimental study of preterm neonates and found that the HCA+AS+ group not developing RDS in the neonatal period shows significantly lower IL-6 in the cord sera compared to the HCA+AS–group not developing RDS, but higher IL-6 than HCA–AS–and HCA–AS+ groups developing RDS, suggesting that the pulmonary protection in HCA+AS+ and HCA+AS–groups is due to fetal lung maturity caused by elevated IL-6. However, IL-6 has been reported to be associated with neurotoxic effects, including IVH and white matter lesions [4,5]. Thereby, *in utero* infection/inflammation is expected to induce dual opposite effects on the developing fetus. In light of hemodynamics in early postnatal period of VLBWIs, it has been reported that chorioamnionitis increases the risk of hypotension [44] whereas AS elevates systemic blood pressure [45].

Taken together, in VLBWIs exposed to both maternal HCA and AS the following mechanisms may be hypothesized. Prenatal inflammation may induce secretion of vasoactive or neurotoxic pro-inflammatory cytokines and may decrease systemic blood pressure in the early postnatal period, with endogenous cortisol secretion as a host defense. Corticosteroids may suppress the pro-inflammatory cytokines and may improve the lowered blood pressure, leading to intermediate cytokine and blood pressure levels, in addition to other benefits of neuroprotection as mentioned above. Accordingly, it is postulated that the cooperation of HCA and AS may complement the harmful weak points of each other to the brain and lead to more stable cerebral vascular environments regarding blood pressure and flow, which may result in protection from IVH related to fluctuating cerebral flow velocity [35] and elevated venous pressure [46]. This may elucidate our most notable result of the combined association of HCA and AS with severe IVH in VLBWIs.

The overall incidence of HCA in the first study population of VLBWIs registered in the KNN was 34.9% (1623 of 4652 infants) (Fig 1). This was higher than the 28.4% in a French nationwide cohort (< 32 weeks’ gestation) [13] and the 31% in an Australian large cohort (< 35 weeks’ gestation) [6], and clearly higher than the reported incidence of clinical CA (17.8% and 15.4%, respectively) in Spanish and Canadian population-based cohorts (< 33 weeks’ gestation) [7,8].

Our study had the strength in that it was based on a nationwide population (up to approximately 70% of VLBWIs in South Korea). In addition, it is the first report to evaluate the combined associations of HCA and AS with neonatal outcomes in VLBWIs via the use of an interaction term. This study also has limitations. The observational nature of the study inherently restricts causal inference despite prospective data were collected under the same strict guidelines of the KNN. Other limitations include potential sources of substantial bias to the results such as physicians’ choices of the use of AS and the relatively high unavailable placental pathology of 16.6%. The lack of data on prenatal antibiotics and the distribution of cases according to the severity and extent of HCA including fetal vasculitis/funicitis should be considered in data interpretation.

In conclusion, our results suggest that in VLBWIs HCA may decrease mortality and AS may reduce mortality and be protective against neonatal seizure while the HCA and AS may work synergistically to prevent severe IVH.
